# Clinical features to distinguish meningitis among young infants at a rural Kenyan hospital

**DOI:** 10.1136/archdischild-2020-318913

**Published:** 2020-08-20

**Authors:** Christina W Obiero, Neema Mturi, Salim Mwarumba, Moses Ngari, Charles Newton, Michael Boele van Hensbroek, James Alexander Berkley

**Affiliations:** 1 Clinical Research Department, KEMRI-Wellcome Trust Research Programme, Kilifi, Kenya; 2 Department of Global Health, University of Amsterdam Faculty of Medicine, Amsterdam, Noord-Holland, The Netherlands; 3 Department of Microbiology, KEMRI-Wellcome Trust Research Programme, Kilifi, Kenya; 4 The Childhood Acute Illness and Nutrition (CHAIN) Network, Nairobi, Kenya; 5 Department of Psychiatry, University of Oxford Centre for Tropical Medicine and Global Health, Oxford, Oxfordshire, UK; 6 Nuffield Department of Medicine, University of Oxford Centre for Tropical Medicine and Global Health, Oxford, Oxfordshire, UK

**Keywords:** general paediatrics, infectious diseases, paediatric practice, tropical infectious disease, tropical paediatrics

## Abstract

**Background:**

Detection of meningitis is essential to optimise the duration and choice of antimicrobial agents to limit mortality and sequelae. In low and middle-income countries most health facilities lack laboratory capacity and rely on clinical features to empirically treat meningitis.

**Objective:**

We conducted a diagnostic validation study to investigate the performance of clinical features (fever, convulsions, irritability, bulging fontanel and temperature ≥39°C) and WHO-recommended signs (drowsiness, lethargy, unconsciousness, convulsions, bulging fontanel, irritability or a high-pitched cry) in discriminating meningitis in young infants.

**Design:**

Retrospective cohort study.

**Setting:**

Kilifi County Hospital.

**Patients:**

Infants aged <60 days hospitalised between 2012 and 2016.

**Main outcome measure:**

Definite meningitis defined as positive cerebrospinal fluid (CSF) culture, microscopy or antigen test, or leucocytes ≥0.05 x 10∧9/L.

**Results:**

Of 4809 infants aged <60 days included, 81 (1.7%) had definite meningitis. WHO-recommended signs had sensitivity of 58% (95% CI 47% to 69%) and specificity of 57% (95% CI 56% to 59%) for definite meningitis. Addition of history of fever improved sensitivity to 89% (95% CI 80% to 95%) but reduced specificity to 26% (95% CI 25% to 27%). Presence of ≥1 of 5 previously identified signs had sensitivity of 79% (95% CI 69% to 87%) and specificity of 51% (95% CI 50% to 53%).

**Conclusions:**

Despite a lower prevalence of definite meningitis, the performance of previously identified signs at admission in predicting meningitis was unchanged. Presence of history of fever improves the sensitivity of WHO-recommended signs but loses specificity. Careful evaluation, repeated assessment and capacity for lumbar puncture and CSF microscopy to exclude meningitis in most young infants with potential signs are essential to management in this age group.

**What is already known on this topic?**

- Meningitis is associated with significant mortality and long-term neurological impairment, particularly in low and middle-income countries where disease burden is highest and diagnostic resources constrained.
- We previously independently identified simple predictors of meningitis (fever, convulsions, irritability, bulging fontanel or temperature ≥39°C) in young infants at our centre.
- Current management guidelines are based on limited evidence obtained prior to use of conjugate vaccines and may not be optimal given changes in disease epidemiology.

**What this study adds?**

- This study investigated the performance of previously identified clinical features and WHO-recommended signs in discriminating meningitis in young infants hospitalised at a rural hospital.
- Meningitis is less common than previously found but performance of clinical features in discriminating meningitis has not changed since the introduction of conjugate vaccines.
- Low specificity of clinical features means that the capacity for basic cerebrospinal fluid analysis is essential to avoid unnecessary treatment.

## Introduction

Meningitis is a life-threatening disease associated with significant mortality and disabling neuropsychological sequelae.^1–4^ Disease burden is highest in low and middle-income countries where about a quarter of children who survive vaccine-preventable meningitis develop postdischarge complications.^5 6^ Prompt recognition and treatment with appropriate antimicrobial coverage and cerebrospinal fluid (CSF) penetration for an adequate duration is critical to optimise outcomes.

CSF culture is the gold standard diagnostic test for meningitis; however, it has limited sensitivity,^7^ is compromised by prior antibiotic exposure^8^ and is frequently unavailable or unreliable in resource-limited hospitals. Changes in CSF cytological and biochemical parameters have diagnostic utility but rarely CSF may be normal in the presence of meningitis.^9 10^ Most health facilities in low and middle-income countries lack CSF diagnostic capacity and so management decisions are based on clinical presentation only.

Young infants typically present to hospital with subtle symptoms and signs,^1 11^ making it challenging for clinicians to decide when to perform a lumbar puncture (LP) (if laboratory facilities exist) or continue empirical antibiotics. The WHO advises suspecting meningitis if an infant: (1) is drowsy, lethargic or unconscious; (2) has convulsions; (3) has a bulging fontanel; (4) is irritable, or (5) has a high-pitched cry.^12^ These guidelines are based on limited evidence collected prior to widespread use of conjugate vaccines,^13^ including a study that reported clinical signs not specific to meningitis.^12^

Between 2001 and 2007, we conducted a study of clinical features associated with meningitis among infants aged <60 days at Kilifi County Hospital (KCH) and found history of fever, convulsions, irritability, bulging fontanel or temperature ≥39°C to be useful indicators of meningitis.^14^ These results, including the WHO recommendation, were incorporated into Kenyan national guidelines.^15^ However, since the introduction of conjugate *Haemophilus influenzae* type b (Hib) vaccine in 2001, an 89% reduction in Hib meningitis in Kenyan children has been observed.^16^ Similarly, 10-valent pneumococcal conjugate vaccine (PCV-10) was introduced in 2011 and was associated with significant reduction in nasopharyngeal carriage of vaccine serotypes,^17^ and incidence and mortality from pneumococcal meningitis.^18–20^ Additionally, the introduction of a voucher scheme and free maternity care in 2013 in Kenya has resulted in a greatly increased number of hospital deliveries and admissions directly to paediatric care.^21^ Although intrapartum antibiotic prophylaxis is recommended in the presence of risk factors for infection such as prolonged rapture of membranes ≥18 hours, maternal screening for group B streptococci (GBS) is not included in the Kenya national guidelines,^22^ despite high incidence of early-onset neonatal infection secondary to GBS.^23^

Thus, changes in both epidemiology and patient profile may have altered the associations between clinical features and meningitis, hence clinical decision rules derived from earlier studies may no longer be optimal. We therefore performed a revalidation study of clinical features at admission to hospital in infants aged <60 days, examining those identified in the previous study at our centre and those recommended by the WHO.

## Methods

### Location and participants

KCH is a government hospital located on the Kenyan coast serving a mostly rural population. Routine vaccination with Hib and PCV-10 vaccines are provided free of charge at government health facilities as a three-dose primary series without a booster dose at 6, 10 and 14 weeks of age. All infants <60 days old hospitalised at KCH between 1 January 2012 and 31 December 2016 were included in this retrospective cohort study.

### Procedures

All infants were systematically assessed by trained clinicians at admission and standardised demographic and clinical data were prospectively collected and entered on a surveillance database in real time. Laboratory investigations done at admission on all infants included haemogram, blood slide for malaria parasites and blood culture. Infants presenting with signs suggestive of meningitis underwent LP and were started on broad-spectrum antibiotics according to WHO^24^ and Kenyan national guidelines.^15^ LP was deferred in infants with cardiorespiratory compromise or signs of raised intracranial pressure.^25^ Infants were assessed daily by clinicians and an LP performed once stable if LP had been delayed at admission, or if an infant developed new clinical features suggestive of meningitis during hospitalisation.

### Laboratory analysis

CSF examination included leucocyte count, Gram and/or Indian ink staining and latex antigen agglutination tests (Wellcogen Bacterial Antigen kit for *Streptococcus pneumoniae*, *H. influenzae*, *Neisseria meningitidis* and CrAg Lateral Flow Assay kit Ref CR2003 for *Cryptococcus neoformans*). All CSF and blood samples were cultured as previously described and pathogens identified using standard methods, including antimicrobial susceptibility testing.^14 26^ Known commensals including coagulase-negative staphylococci were considered non-significant. CSF protein and glucose and concurrent blood glucose were measured on an Instrument Laboratory Aries analyser (Werfen, Germany).

Sample processing and analysis was performed at the KEMRI Centre for Geographic Medicine (Coast) laboratory which is externally monitored for quality assurance by the UK External Quality Assessment Service and accredited in Good Clinical Laboratory Practice by Qualogy, UK.

### Definitions

For this analysis, we defined definite meningitis according to the criteria used in our previous study^14^: (1) positive CSF culture for a known pathogen; or (2) organisms observed on CSF Gram stain microscopy; or (3) positive CSF antigen test; or (4) CSF leucocytes ≥0.05 x 10∧9/L50 cells/µL. Possible meningitis was defined in infants without definite meningitis as: CSF leucocytes ≥0.02 x 10∧9/L in infants aged 0–28 days, and CSF leucocytes ≥0.01 x 10∧9/L in infants aged 28–59 days. Infants not meeting either criteria were defined as no meningitis. Possible meningitis and the narrow microbiological criteria for definite meningitis (positive CSF culture, antigen test or microscopy, or CSF leucocytes ≥0.05 x 10∧9/L plus positive blood culture) were used for sensitivity analysis.

### Statistical analysis

We extracted data from the surveillance database. Infants who died before an LP had been performed were then excluded as we could not ascertain their meningitis status. We analysed data from all infants and then separately examined those 0–6 days and 7–59 days because of potential differences in aetiology and clinical presentation.^14^

We calculated the prevalence of meningitis and tabulated the frequency distribution of CSF findings, including pathogens identified and the highest CSF criterion for definite meningitis attained in the order of the four criteria given above.

We examined the performance of the previously identified clinical features (history of fever, convulsions, irritability, bulging fontanel and temperature ≥39°C)^14^ and the WHO-recommended signs^24^ by calculating their sensitivity, specificity, positive predictive value (PPV), negative predictive value (NPV) and area under receiver operating characteristic (ROC) curve for definite meningitis versus no meningitis.

We calculated the number of LPs needed to identify one case of definite meningitis using each combination of features as the inverse of the risk difference obtained by subtracting the risk of meningitis in the group with indicator(s) of interest from risk of meningitis in the group without indicator(s) of interest. As a sensitivity analysis we then repeated these analyses for possible or definite meningitis versus no meningitis.

Proportions were compared using the χ^2^ test or Fisher’s exact test, while continuous variables were compared using Wilcoxon rank-sum test. We performed analyses using Stata V.15 (StataCorp, USA).

## Results

**Figure 1:**
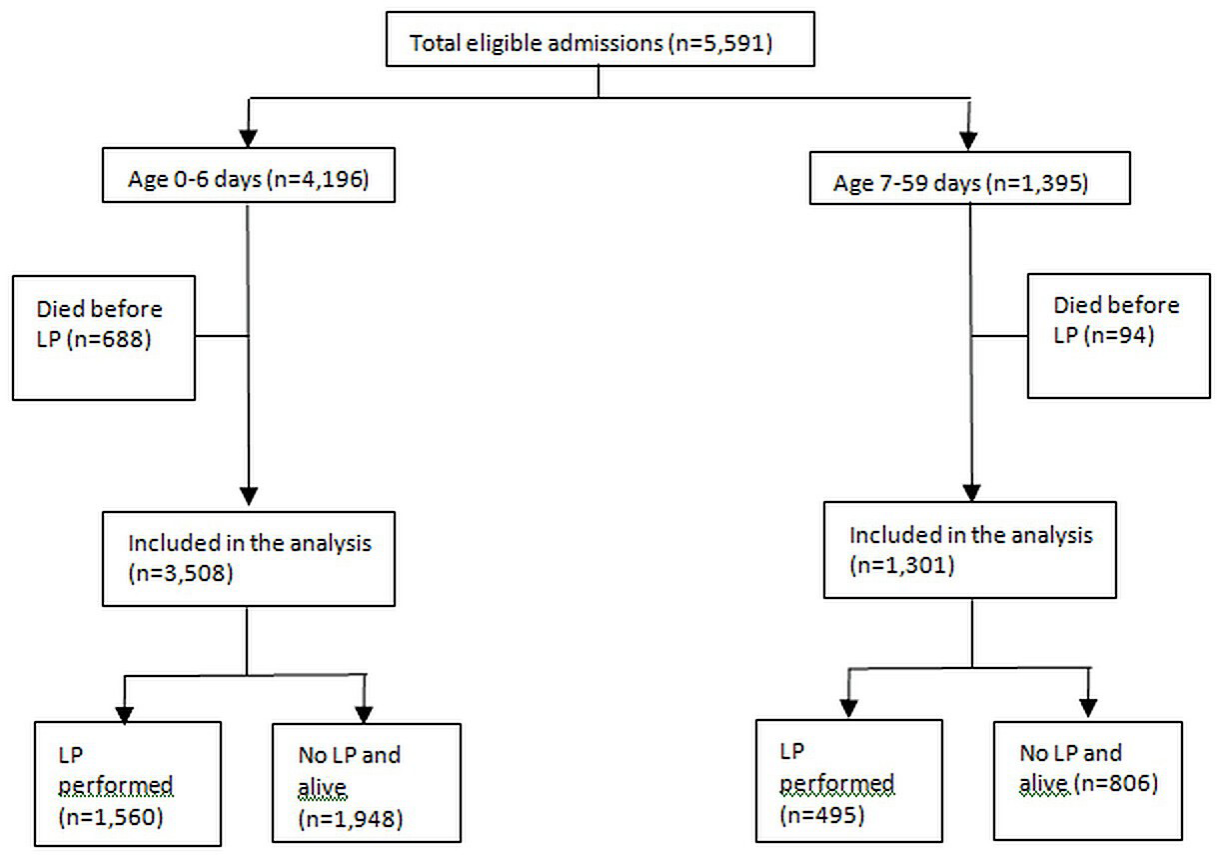
Flow chart of study participants. LP, lumbar puncture.

During the study period, 5591 infants were admitted, of which 4196 (75%) were aged 0–6 days. Three thousand two hundred and fifty-three (58%) infants were born at KCH, including 2747 (65%) of those aged 0–6 days of whom 2476 (90%) were hospitalised within the first 72 hours of life. Overall, 853/5591 (15%) infants died during hospitalisation and 640/853 (75%) deaths occurred during the first day of life. Seven hundred and eighty-two (92%) of 853 deaths occurred before an LP had been performed and were excluded from this analysis (figure 1). Thus, 4809 infants of which 3508 (73%) were aged 0–6 days were included in the analysis.

Eighty-one (1.7%) infants had definite meningitis; 39/3508 (1.1%) aged 0–6 days and 42/1301 (3.2%) aged 7–59 days (p<0.001). Eighteen (22%) infants had positive CSF culture (table 1) of which eight had a positive blood culture and six grew similar organisms (four GBS, one group A streptococcus and one *Escherichia coli*) in blood and CSF. GBS was the most common CSF isolate followed by *E. coli* and *Klebsiella pneumoniae*. Two infants had Hib and one had pneumococcal meningitis; none had positive CSF or blood cultures. Twenty-seven (33%) of the 81 definite meningitis cases had visibly turbid CSF. Three of 11 infants with positive blood culture had CSF leucocytes ≥0.05 x 10∧9/L. Seven (8.6%) infants with definite meningitis died during admission compared with 64 (1.4%) in the non-meningitis group, p<0.001. Online supplementary table S1 shows the meningitis cases and the number of LPs during our study period versus our previous analysis.

10.1136/archdischild-2020-318913.supp1 Supplementary data

**Table 1:** Diagnostic criteria for meningitis and bacterial species detected

Diagnostic criteria	Ages 0–6 days (3508/4809, 73%)	Ages 7–59 days (1301/4809, 27%)	Total positives	Highest criteria for meningitis
CSF culture				
Gram positive				
Group B streptococci	0	7	7*	7
Group A streptococci	0	1	1†	1
Gram negative				
*Escherichia coli*	2	3	5‡	5
*Klebsiella pneumonia*	1	1	2§	2
*Klebsiella oxytoca*	0	1	1	1
*Enterobacter aerogenes*	0	1	1	1
*Citrobacter* sp	1	0	1¶	1
Total	4	14	18	18
Latex antigen test				
*Streptococcus pneumoniae*	0	1	1**	1
*Haemophilus influenzae*	1	1	2††	2
*Cryptococcus neoformans*	1	0	1	1
Total positive antigen test	2	2	4	4
Gram stain				
Gram-positive cocci	0	6	6	0
Gram-negative rods	5	1	6	2
Total	5	7	12‡‡	2
Indian ink	1	0	1	1
CSF WCC ≥0.05 x 10∧9/L	33	37	70	56
Total				81

*6/7 had WCC ≥0.05 x 10∧9/L and 5/7 had positive Gram stain.

†Had WCC ≥0.05 x 10∧9/L and positive Gram stain.

‡1/5 had *Streptococcus* sp isolated as well; 3/5 had WCC ≥0.05 x 10∧9/L; 2/5 had positive Gram stain.

§1/2 had WCC ≥0.05 x 10∧9/L; one had positive Gram stain.

¶Had WCC ≥0.05 x 10∧9/L and positive Gram stain.

**Had WCC ≥0.05 x 10∧9/L.

††1/2 had WCC ≥0.05 x 10∧9/L.

‡‡10/12 had positive CSF culture; 9/10 had WCC ≥0.05 x 10∧9/L.

CSF, cerebrospinal fluid; WCC, white cell count.

### Clinical features at admission

History of fever, bulging fontanel, axillary temperature ≥39°C and irritability were associated with definite meningitis among infants aged 0–6 days (table 2). Bulging fontanel, convulsions and irritability were associated with definite meningitis in older infants. A bulging fontanel, stiff neck and inability to breast feed were each observed in only 5% of meningitis cases.

**Table 2:** Clinical history and examination findings among neonates and young infants

Characteristic	Ages 0–6 days			Ages 7–59 days		
	No meningitis (n=3469)	Meningitis (n=39)	P value*	No meningitis (n=1259)	Meningitis (n=42)	P value*
Bulging fontanel						
No	3419 (99)	37 (95)	<0.001	1233 (98)	33 (79)	<0.001
Yes	15 (0.4)	2 (5.1)		11 (0.9)	9 (21)	
Missing	35 (1.0)	0 (0)		15 (1.2)	0 (0)	
Convulsions						
No	3269 (94)	34 (87)	0.063	1162 (92)	31 (74)	<0.001
Yes	168 (4.8)	5 (13)		82 (6.5)	11 (26)	
Missing	32 (0.9)	0 (0)		15 (1.2)	0 (0)	
Axillary temperature (°C)						
<36	1061 (31)	4 (10)	0.021	77 (6.1)	1 (2.4)	0.269
36–38.9	2202 (63)	30 (77)		1092 (87)	35 (83)	
≥39	199 (5.7)	5 (13)		89 (7.1)	6 (14)	
Missing	7 (0.2)	0 (0)		1 (0.1)	0 (0)	
Agitation/irritability						
No	3375 (97)	33 (85)	<0.001	1198 (95)	36 (86)	0.002
Yes	59 (1.7)	6 (15)		46 (3.7)	6 (14)	
Missing	35 (1.0)	0 (0)		15 (1.2)	0 (0)	
History of fever†						
No	2086 (60)	14 (36)	0.006	470 (37)	9 (21)	0.074
Yes	1350 (39)	25 (64)		774 (61)	33 (79)	
Missing	33 (1.0)	0 (0)		15 (1.1)	0 (0)	
Drowsy, lethargic or unconscious						
No	2626 (76)	27 (69)	0.466	1048 (83)	31 (74)	0.148
Yes	808 (23)	12 (31)		196 (16)	11 (26)	
Missing	35 (1.0)	0 (0)		15 (1.2)	0 (0)	
Abnormal cry						
No	2489 (72)	33 (85)	0.093	846 (67)	31 (74)	0.587
Yes	945 (27)	5 (13)		118 (9.4)	4 (9.5)	
Missing	35 (1.0)	1 (2.6)		295 (23)	7 (!7)	

Data are n (%).

Group percentages may not add to 100% due to rounding off.

*Univariate comparison of characteristics.

†Elevated tactile temperature as reported by the parent or guardian.

### Performance of clinical features in all infants

#### Previously identified signs

Sixty-four (2.7%) of 2377 infants presenting with one or more of history of fever, irritability, axillary temperature ≥39°C, convulsions or bulging fontanel had definite meningitis compared with 17/2432 (0.7%) infants lacking these features (p<0.001): sensitivity 79% (95% CI 69% to 87%), specificity 51% (95% CI 50% to 53%), PPV 2.7% (95% CI 2.1% to 3.4%), NPV 99% (95% CI 99% to 100%). Fifty infants (95% CI 37 to 79) presenting with one or more of these clinical features would need to undergo an LP for each case of meningitis to be identified (table 3).

**Table 3:** Performance of indicators of meningitis among all infants 0–59 days old

Indicators	Number with indicator	Number with meningitis	Sensitivity (95% CI)	Specificity (95% CI)	PPV (95% CI)	NPV (95% CI)	NN LP (95% CI)
**Previously identified**							
Bulging fontanel	37	11	13.6 (7.0 to 23.0)	99.4 (99.2 to 99.6)	29.7 (15.9 to 47.0)	98.5 (98.1 to 98.8)	4 (2 to 7)
Convulsions or any of the above	292	24	29.6 (20.0 to 40.8)	94.3 (93.6 to 95.0)	8.2 (5.3 to 12.0)	98.7 (98.4 to 99.0)	14 (10 to 26)
Axillary temperature ≥39°C or any of the above	555	32	39.5 (28.8 to 51.0)	88.9 (88.0 to 89.8)	5.8 (4.0 to 8.0)	98.8 (98.5 to 99.1)	22 (15 to 38)
Agitation/irritability or any of the above	640	37	45.7 (34.6 to 57.1)	87.2 (86.3 to 88.2)	5.8 (4.1 to 7.9)	98.9 (98.6 to 99.2)	21 (15 to 35)
History of fever or any of the above	2377	64	79.0 (68.5 to 87.3)	51.1 (49.6 to 52.5)	2.7 (2.1 to 3.4)	99.3 (98.9 to 99.6)	50 (37 to 79)
**WHO recommended**							
One or more of the WHO-suggested signs	2072	47	58.0 (46.5 to 68.9)	57.2 (55.7 to 58.6)	2.3 (1.7 to 3.0)	98.8 (98.3 to 99.1)	98 (56 to 381)
One or more of the WHO-suggested signs or history of fever	3566	72	88.9 (80.0 to 94.8)	26.1 (24.9 to 27.4)	2.0 (1.6 to 2.5)	99.3 (98.6 to 99.7)	77 (51 to 157)

NN LP, number needed to lumbar puncture to identify one case of definite meningitis; NPV, negative predictive value; PPV, positive predictive value.

#### WHO-recommended signs or a history of fever

Forty-seven (2.3%) of 2072 infants presenting with one or more of WHO-recommended signs had definite meningitis compared with 34/2737 (1%) infants lacking these signs (p=0.006): sensitivity 58% (95% CI 47% to 69%), specificity 57% (95% CI 56% to 59%), PPV 2.3% (95% CI 1.7% to 3%) and NPV 99% (95% CI 98% to 99%). Ninety-eight infants (95% CI 56 to 381) presenting with one or more of WHO-recommended signs would need to undergo an LP for each meningitis case to be identified (table 3). Addition of history of fever to these WHO signs resulted in sensitivity 89% (95% CI 80% to 95%), specificity 26% (95% CI 25% to 27%), PPV 2% (95% CI 1.6% to 2.5%) and NPV 99% (95% CI 99% to 100%).

### Clinical features in infants 0–6 days old compared with infants 7–59 days old

Previously identified signs were less sensitive but more specific in detecting meningitis in infants in the first week of life than among infants 7–59 days old (online supplementary tables S2 and S3). WHO-recommended signs had similar sensitivity and specificity in both age groups. History of fever markedly improved the sensitivity of WHO-recommended signs but resulted in low specificity in both age groups.

**Figure 2:**
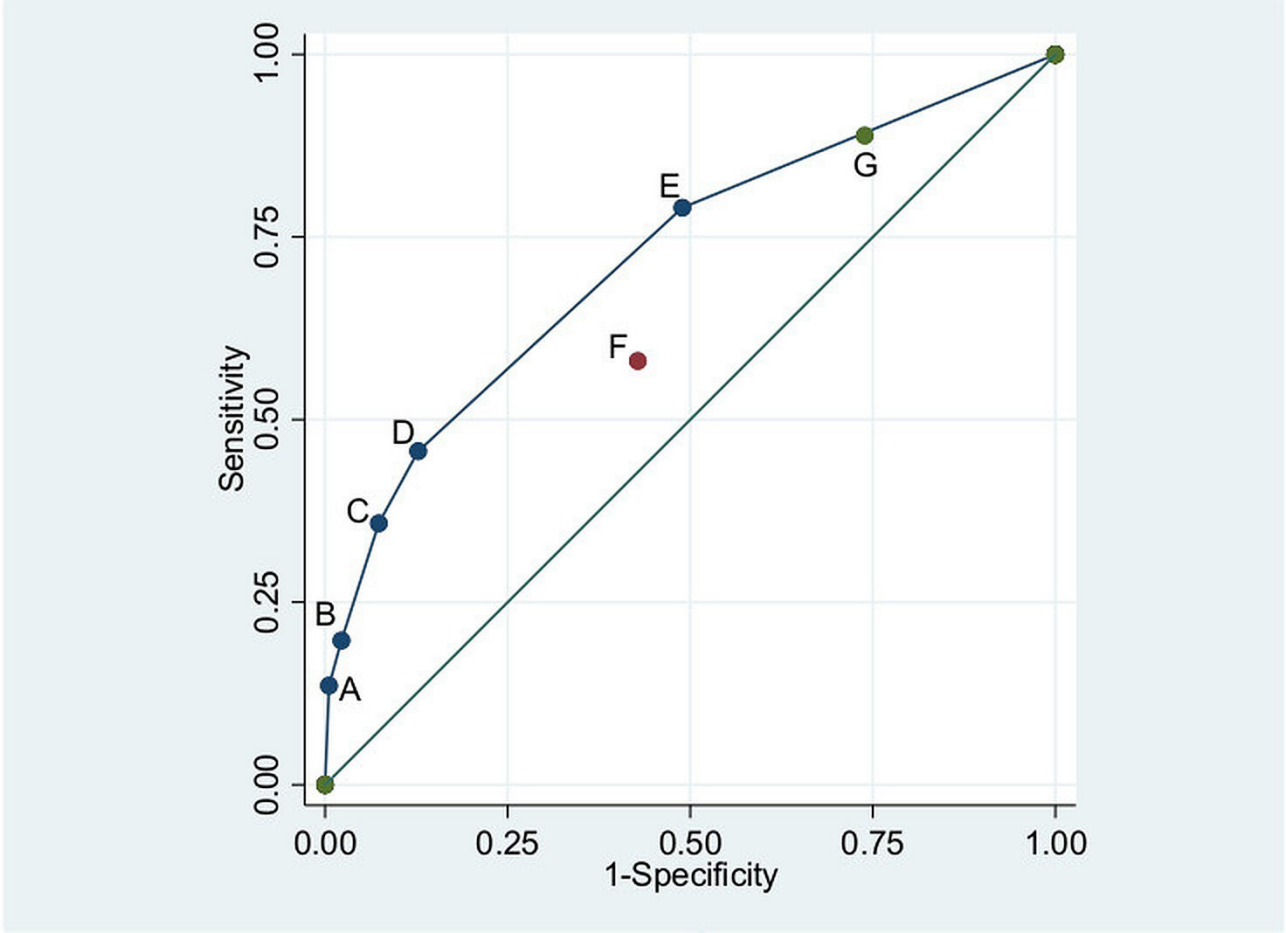
Area under receiver operating characteristic (ROC) curve for previously identified signs and WHO-recommended signs of meningitis or history of fever in infants aged 0–59 days. (A) Bulging fontanel. (B) Convulsions. (C) Axillary temperature ≥39°C. (D) Agitation/irritability. (E) History of fever. (F) WHO-recommended signs. (G) WHO-recommended signs or history of fever.

The overall area under the ROC curve for previously identified signs was 0.72 (95% CI 0.66 to 0.78) and there was no evidence that this differed between infants aged 0–6 days and aged 7–59 days (p=0.19) (figure 2).

### Sensitivity analysis

One hundred and twenty-six infants had possible or definite meningitis. Fewer LPs were needed to identify a single case of possible or definite meningitis compared with a cut-off ≥50/µL in all infants (online supplementary table S4). The overall area under the ROC curve for previously identified signs was 0.69 (95% CI 0.64 to 0.74) and there was no evidence that this differed between infants aged 0–6 days and aged 7–59 days (p=0.17).

For the 28 (0.6%) infants with definite meningitis based on microbiological criteria, point estimates for sensitivity and specificity for previously identified signs, and WHO-recommended signs or a history of fever respectively were similar to the main analysis and more LPs were needed to identify a single case of meningitis (online supplementary table S5).

## Discussion

This study aimed to determine if clinical features at admission to hospital that were found to discriminate young infants with meningitis before widespread use of conjugate vaccines in low and middle-income countries are still applicable for decision-making. Overall, 1.7% infants included in our study had definite meningitis, lower than 4.2% in 2006–2007 and 4.1% in 2001–2005 previously at our centre using the same definition and inclusion criteria.^14^ The number of infants hospitalised at KCH has increased, predominantly due to increased admissions on the first day of life related to uptake of free maternity care.^14 21^ Meningitis cases decreased with time despite an increase in the number of LPs performed. Comparing the present study (2012–2016) to our previous study (1994–1998),^27^ there were 1 vs 24 cases of *S. pneumoniae* and 2 vs 11 cases of *H. influenzae* meningitis demonstrating an effect of herd immunity from conjugate vaccination on case load.

In addition to limited CSF diagnostic capacity, ancillary tests that may be helpful in stratifying infants at risk of meningitis such as peripheral blood leucopenia,^28^ absolute neutrophilia^29^ and biomarkers (eg, procalcitonin)^30^ are usually unavailable and have not been validated in our setting. This further underscores the need to optimise clinical guidelines to identify infants needing urgent treatment.

While the presence of one or more of the previously identified signs missed less cases than WHO-recommended signs alone (21% vs 42%), history of fever improved the performance of WHO signs. Our results were similar to those reported by our previous study,^14^ suggesting that although meningitis is now less common, performance of these signs, including those recommended by WHO, has not significantly changed over time. Of importance, none of these signs were highly specific in discriminating meningitis at admission, hence careful clinical review and LP are essential, especially in consideration of duration of antibiotics once started.

Capacity for CSF analysis is often unavailable in resource-limited settings^31^ and more LPs are now needed to identify a single meningitis case than previously, especially with the narrowest meningitis case definition.^14^ There are limited data on the ‘acceptable’ number of LPs needed to diagnose a single case of meningitis in young infants, with most studies focusing on the clinical utility of LPs in older children with seizures.^32^

Typically, about 50% meningitis deaths occur within the first 24 hours of admission and postmortem LP may be useful for surveillance and studies of aetiology,^27^ but undertaking LP promptly is vital for diagnosis. The low specificity of clinical signs leads to overdiagnosis. However, given the high risk of morbidity and mortality associated with bacterial meningitis and the contribution to development of antimicrobial resistance of overtreatment, any reluctance to perform LPs, even where full laboratory support is unavailable, needs to be addressed as failing to do so will miss 10%–40% of cases and/or overtreat the vast majority of infants with indicator signs (online supplementary table S4). Meningitis is now less common, and more difficult to exclude purely on clinical grounds and clinicians should maintain a low threshold for doing LPs, such as in all infants with fever in addition to more specific signs, especially in 0–6 day-olds.

Thirty seven per cent of meningitis cases in our study had turbid CSF, similar to 28% cases in 2001–2005 (p=0.18). CSF leucocyte count may fail to discriminate infants with culture-proven meningitis from those without,^10^ but together with visual turbidity would have identified 71 (88%) of definite cases and 118 (94%) of possible cases. Sensitivity analysis done in our study with a lower cut-off (≥20/µL), which has been shown to provide sufficient diagnostic precision for culture-proven meningitis,^7^ did not alter our results. Support to establish basic CSF cell counting and Gram stain in resource-limited settings to optimise antimicrobial treatment is essential to providing inpatient paediatric services.

### Limitations

We lacked data on prehospital antibiotic exposure which has been shown to lower CSF culture yield.^8 33^ Potential misclassification of infants with negative CSF findings or asymptomatic infants may have diminished the validity of clinical features studied. Infants who died prior to an LP were excluded from this analysis, however, we did not aim to estimate the overall burden of meningitis, rather to address the challenges faced in clinical practice among infants in whom a decision to admit to hospital had been made. Delays in presentation to hospital^34^ and rapid disease progression may have led to early mortality before LP. This may have a pathogen-specific impact on our findings as, for example, GBS mortality commonly occurs in the first few hours of life.^23^

## Conclusions

Meningitis is an uncommon but important diagnosis in young infants. Despite declining incidence, clinical features of meningitis do not perform less well now than in the preconjugate vaccine era. However, clinicians and policymakers should be aware of the number of LPs or empirical treatments needed for each case of definite meningitis identified. The clinical signs currently recommended by WHO to guide decisions to perform an LP and initiate antibiotics poorly discriminate infants with meningitis, particularly in neonates aged <1 week. History of fever is an important indicator and clinicians should not rely on ‘classical’ signs such as neck stiffness or bulging fontanel only. Even the best-performing clinical decision rule fails to identify all cases when applied at admission and has poor specificity, hence it is important that all young infants hospitalised with serious illness undergo an LP.
