# Supplementary material for: Clinical features to distinguish meningitis among young infants at a rural Kenyan hospital
Source: Arch Dis Child. 2020 Aug 20;106(2):130–6. doi: 10.1136/archdischild-2020-318913 (PMC7841476; doi:10.1136/archdischild-2020-318913)
Supplement: Supplementary data [file archdischild-2020-318913supp001.pdf]

Clinical features to distinguish meningitis among young infants at a rural Kenyan hospital

Supplementary material

Contents

Clinical features to distinguish meningitis among young infants at a rural Kenyan hospital................ 0

Supplementary material.. 0

Table S1. Comparison of annual admissions, lumbar punctures and meningitis cases during our study period and our previous analysis.. 1

Table S2. Performance of indicators of meningitis among neonates 0-6 days old.......................... 2

Table S3. Performance of indicators of meningitis among neonates 7-59 days old........................ 3

Table S4. Performance of indicators of meningitis among all infants 0-59 days old, including possible meningitis[a] 4

Table S5. Performance of indicators of meningitis among all infants 0-59 days old using microbiologically confirmed definite meningitis[a].. 5

Table S1. Comparison of annual admissions, lumbar punctures and meningitis cases during our study period and our previous analysis

Study period	2012-2016	2006-2007	2001-2005
Admissions/year	1,118	839	690
Lumbar punctures/year	411	517	210
Meningitis cases/year	16	32	20
Data are N			

Obiero CW, *et al. Arch Dis Child* 2021; 106:130–136. doi: 10.1136/archdischild-2020-318913

Table S2. Performance of indicators of meningitis among neonates 0-6 days old

**Indicators**	**Number with indicator**	**Number with meningitis**	**Sensitivity (95% CI)**	**Specificity (95% CI)**	**PPV (95% CI)**	**NPV (95% CI)**	**NNLP (95% CI)**
Bulging fontanel	17	2	5.1 (0.6-17.3)	99.6 (99.3-99.8)	11.8 (1.5-36.4)	98.9 (98.5-99.2)	9 (4 - -22)
Convulsions or any of the above	184	7	17.9 (7.5-33.5)	94.9 (94.1-95.6)	3.8 (1.5-7.7)	99.0 (98.6-99.3)	35 (18-1,734)
Axillary temp ≥39ºC or any of the above	365	11	28.2 (15.0-44.9)	89.8 (88.7-90.8)	3.0 (1.5 (5.3)	99.1 (98.7-99.4)	47 (26-296)
Agitation/irritability or any of the above	414	15	38.5 (23.4-55.4)	88.5 (87.4-89.5)	3.6 (2.0-5.9)	99.2 (98.8-99.5)	35 (21-98)
History of fever or any of the above	1,509	26	66.7 (49.8-80.9)	57.2 (55.6-58.9)	1.7 (1.1-2.5)	99.3 (98.9-99.7)	93 (55-305)
One or more of the WHO suggested signs	1,598	22	56.4 (39.6-72.2)	54.6 (52.9-72.2)	1.4 (0.9-2.1)	99.1 (98.6-99.5)	206 (84- -448)
One or more of the WHO suggested signs or history of fever	2,573	31	79.5 (63.5-90.7)	26.7 (25.3-28.2)	1.2 (0.8-1.7)	99.1 (98.3-99.6)	286 (93- -266)

Abbreviations: CI, confidence interval; PPV, positive predictive value; NPV, negative predictive value; NNLP, number needed to lumbar puncture

BMJ Publishing Group Limited (BMJ) disclaims all liability and responsibility arising from any reliance placed on this supplemental material which has been supplied by the author(s)

Table S3. Performance of indicators of meningitis among neonates 7-59 days old

**Indicators**	**Number with indicator**	**Number with meningitis**	**Sensitivity (95% CI)**	**Specificity (95% CI)**	**PPV (95% CI)**	**NPV (95% CI)**	**NNLP (95% CI)**
Bulging fontanel	20	9	21.4 (10.3-36.8)	99.1 (98.4-99.6)	45.0 (23.1-68.5)	97.4 (96.4-98.2)	2 (2-5)
Convulsions or any of the above	108	17	40.5 (25.6-56.7)	92.8 (91.2-94.1)	15.7 (9.5-24.0)	97.9 (96.9-98.6)	7 (5-15)
Axillary temp ≥39°C or any of the above	190	21	50.0 (34.2-65.8)	86.6 (84.6-88.4)	11.1 (7.0-16.4)	98.1 (97.1-98.8)	11 (7-22)
Agitation/irritability or any of the above	226	22	52.4 (36.4-68.0)	83.8 (81.6-85.8)	9.7 (6.2-14.4)	98.1 (97.1-98.9)	13 (9-26)
History of fever or any of the above	868	38	90.5 (77.4-97.3)	34.1(31.5-36.8)	4.4 (3.1-6.0)	99.1 (97.7-99.7)	29 (20-55)
One or more of the WHO suggested signs	474	25	59.5 (43.3-74.4)	64.3 (61.6-67.0)	5.3 (3.4-7.7)	97.9 (96.7-98.8)	31 (18-101)
One or more of the WHO suggested signs or history of fever	993	41	97.6 (87.4-99.9)	24.4 (22.0-26.9)	4.1 (3.0-5.6)	99.7 (98.2-100)	26 (19-41)

Abbreviations: CI, confidence interval; PPV, positive predictive value; NPV, negative predictive value; NNLP, number needed to lumbar puncture

Obiero CW, *et al. Arch Dis Child* 2021; 106:130–136. doi: 10.1136/archdischild-2020-318913

Table S4. Performance of indicators of meningitis among all infants 0-59 days old, including possible meningitis[a]

Indicators	Number with indicator	Number with meningitis	Sensitivity (95% CI)	Specificity (95% CI)	PPV (95% CI)	NPV (95%CI)	NNLP (95% CI)
Bulging fontanel	37	14	11.1 (6.2-17.9)	99.5 (99.3-99.7)	37.8 (22.5-55.2)	97.6 (97.2-98.0)	3 (2-5)
Convulsions or any of the above	292	36	28.6 (20.9-37.3)	94.5 (93.8-95.2)	12.3 (8.8-16.7)	98.0 (97.6-98.4)	10 (7-15)
Axillary temp ≥39°C or any of the above	555	46	36.5 (28.1-45.6)	89.1 (88.2-90.0)	8.3 (6.1-10.9)	98.1 (97.7-98.5)	16 (11-25)
Agitation/irritability or any of the above	640	52	41.3 (32.6-50.4)	87.4 (86.5-88.4)	8.1 (6.1-10.5)	98.2 (97.8-98.6)	16 (12-24)
History of fever or any of the above	2,377	94	74.6 (66.1-81.9)	51.2 (49.8-52.7)	4.0 (3.2-4.8)	98.7 (98.1-99.1)	38 (28-58)
One or more of the WHO suggested signs	2,072	77	61.1 (52.0-69.7)	57.4 (56.0-58.8)	3.7 (2.9-4.6)	98.2 (97.6-98.7)	52 (35-103)
One or more of the WHO suggested signs or history of fever	3,566	113	89.7 (83.0-94.4)	26.3 (25.0-27.6)	3.2 (2.6-3.8)	99.0 (98.2-99.4)	47 (34-76)

Abbreviations: CI, confidence interval; PPV, positive predictive value; NPV, negative predictive value; NNLP, number needed to lumbar puncture

[a] Possible meningitis defined in infants without definite meningitis as CSF WBC ≥20/µL and <50/µL in infants age 0-28 days, or CSF WBC ≥10/µL and <50/µL in infants age 29-59 days.

Table S5. Performance of indicators of meningitis among all infants 0-59 days old using microbiologically confirmed definite meningitis[a]

Indicators	Number with indicator	Number with meningitis	Sensitivity (95% CI)	Specificity (95% CI)	PPV (95% CI)	NPV (95%CI)	NNLP (95% CI)
**Previously identified**							
Bulging fontanel	37	6	21.4 (8.3-41.0)	99.3 (99.1-99.6)	16.2 (6.2-32.0)	99.5 (99.3-99.7)	6 (4-26)
Convulsions or any of the above	292	8	28.6 (13.2-48.7)	94.1 (93.4-94.7)	2.7 (1.2-5.3)	99.6 (99.3-99.7)	44 (24-241)
Axillary temp ≥39°C or any of the above	555	14	50.0 (30.6-69.4)	88.7 (87.8-89.6)	2.5 (1.4-4.2)	99.7 (99.4-99.8)	46 (28-114)
Agitation/irritability or any of the above	640	15	53.6 (33.9-72.5)	86.9 (85.9-87.9)	2.3 (1.3-3.8)	99.7 (99.5-99.8)	49 (31-118)
History of fever or any of the above	2,377	24	85.7 (67.3-96.0)	50.8 (49.4-52.2)	1.0 (0.6-1.5)	99.8 (99.6-100.0)	118 (78-243)
**WHO recommended**							
One or more of the WHO suggested signs	2,072	15	53.6 (33.9-72.5)	57.0 (55.6-58.4)	0.7 (0.4-1.2)	99.5 (99.2-99.7)	402 (144-506)
One or more of the WHO suggested signs or history of fever	3,566	24	85.7 (67.3-96.0)	25.9(24.7-27.2)	0.7 (0.4-1.0)	99.7 (99.2-99.9)	285 (131-1,601)

Abbreviations: CI, confidence interval; PPV, positive predictive value; NPV, negative predictive value; NNLP, number needed to lumbar puncture to identify one case of definite meningitis
[a]Includes positive CSF culture, positive latex agglutination test, positive microscopy, or CSF leukocyte count ≥50 cells/µL plus positive blood culture
